# Atomic Force Microscope nanolithography on chromosomes to generate single-cell genetic probes

**DOI:** 10.1186/1477-3155-9-27

**Published:** 2011-06-28

**Authors:** Sebastiano Di Bucchianico, Anna M Poma, Maria F Giardi, Luana Di Leandro, Francesco Valle, Fabio Biscarini, Dario Botti

**Affiliations:** 1 Department of Basic and Applied Biology, University of L'Aquila, Via Vetoio 1, L'Aquila 67100, Italy; 2 Institute for Nanostructured Materials, Consiglio Nazionale delle Ricerche ISMN-CNR, Via P. Gobetti 101, Bologna 40129, Italy

## Abstract

**Background:**

Chromosomal dissection provides a direct advance for isolating DNA from cytogenetically recognizable region to generate genetic probes for fluorescence in situ hybridization, a technique that became very common in cyto and molecular genetics research and diagnostics. Several reports describing microdissection methods (glass needle or a laser beam) to obtain specific probes from metaphase chromosomes are available. Several limitations are imposed by the traditional methods of dissection as the need for a large number of chromosomes for the production of a probe. In addition, the conventional methods are not suitable for single chromosome analysis, because of the relatively big size of the microneedles. Consequently new dissection techniques are essential for advanced research on chromosomes at the nanoscale level.

**Results:**

We report the use of Atomic Force Microscope (AFM) as a tool for nanomanipulation of single chromosomes to generate individual cell specific genetic probes. Besides new methods towards a better nanodissection, this work is focused on the combination of molecular and nanomanipulation techniques which enable both nanodissection and amplification of chromosomal and chromatidic DNA. Cross-sectional analysis of the dissected chromosomes reveals 20 nm and 40 nm deep cuts. Isolated single chromosomal regions can be directly amplified and labeled by the Degenerate Oligonucleotide-Primed Polymerase Chain Reaction (DOP-PCR) and subsequently hybridized to chromosomes and interphasic nuclei.

**Conclusions:**

Atomic force microscope can be easily used to visualize and to manipulate biological material with high resolution and accuracy. The fluorescence *in situ *hybridization (FISH) performed with the DOP-PCR products as test probes has been tested succesfully in avian microchromosomes and interphasic nuclei. Chromosome nanolithography, with a resolution beyond the resolution limit of light microscopy, could be useful to the construction of chromosome band libraries and to the molecular cytogenetic mapping related to the investigation of genetic diseases.

## Background

The conventional approach to chromosomes microdissection is based on the use of thin glass needles for the collection of chromosomes and chromosomal regions. The number of copies of dissected chromosomes needed for the generation of painting probes, varies from more than 50 [1] to less than 10 [2]. A modified protocol which reduces the copy number of microdissected DNA fragments has been developed by laser pressure catapulting and amplification using linker-adaptor PCR [3]. Chromosome recognition is a prerequisite of this technique so the chromosome microdissection method was widely used in genomics research correlated to the G-banding technique.

Since its development in 1986 by Binnig et al [4], the AFM has played a crucial role in the nanoscale biomedical research [5,6]. The AFM is a microscopic system that generates a surface topography by using attractive and repulsive interaction forces between a sharp Si or SiO2 tip attached to a cantilever and a sample. By approaching the cantilever to the sample, the interaction forces can be measured and controlled; upon scanning the surface it will thus be possible to record the topography of the sample. This features allow the AFM to work on unstained and uncoated chromosomes [7]. The AFM imaging reveals that the chromosomes are not uniform in structure but have, along their length, ridges and grooves that may be related to the G-positive and G-negative bands respectively [8,9]. In this way it is possible to recognize and manipulate chromosomal regions without staining and coating.

Cytogenetic analysis of MDCC-MSB1, a chicken T-cell line transformed with Marek's Disease Virus (MDV), has been performed with both classical methods and AFM demonstrating a duplication of the short arm of chromosome 1, (1p)(p22-p23) [10].

It must be underlined that the chicken karyotype consist of 39 chromosomes, 30 of which are classed as microchromosomes (MICs) and are cytologically impossible to differentiate from each other because of their small size [11]. For this reason it is interesting to use the AFM as a tool to manipulate chromosomes and to generate probes for fluorescence in situ hybridization (FISH), confirming the duplication of chromosome 1 and making the microchromosomes univocally recognizable. The generation of chromosomal painting probes from a single unstained chromosome or a single chromosomal region can be helpful in studies focusing on comparative genomics and genomic organization, as well as in clinical diagnostic of mosaicisms or in heterogeneous cell populations.

Here, we describe the production of specific painting probes from a single avian microchromosome and a single chromosomal region using the AFM. When an increasing force is applied to the microscope tip, a nanosize chromosomal region can be dissected away, collecting DNA fragment adherent to the tip. We introduce nanolithography on chromosomes surface where contiguous line patterns can be generated by a software-controlled pattern generator built in the AFM controller. Controlling the lithography software the tip can be moved with a specified speed along the precise scanning lines. The nanodissected DNA can be amplified through DOP-PCR [12].

## Results

**Figure 1:**
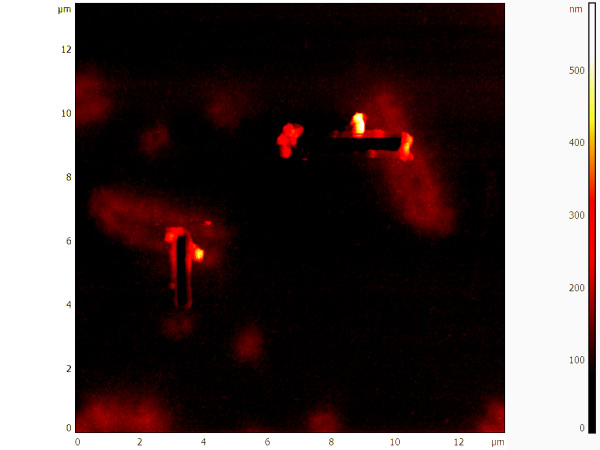
**Topographic AFM micrograph of chromosomes 3 after DNA extraction**. Upon localization of the chromosome region to be dissected the AFM microscope is switched in Contact Nanolithography Mode and the probe is scanned at high force (5-10 μN) several time for few lines (up to 8) perpendicularly to the chromatide. The cross sectional analysis of the cut site reveals a full width at half-maximum height of around 50 nm.

**Figure 2:**
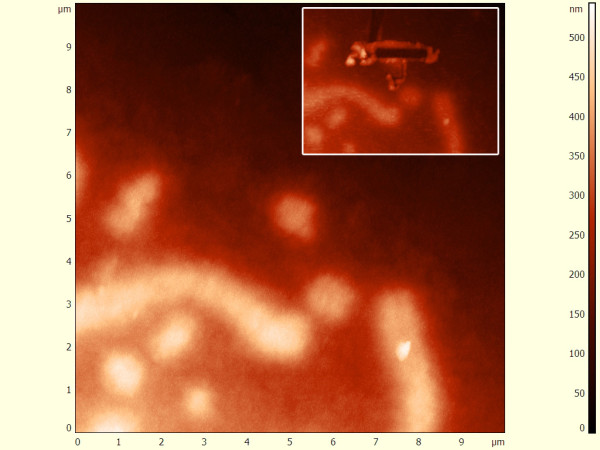
**Topographic AFM micrograph of microchromosome**. Microchromosome before and after (insert) DNA extraction. The cross sectional analysis of the cut site reveals a full width at half-maximum height of around 40 nm.

**Figure 3:**
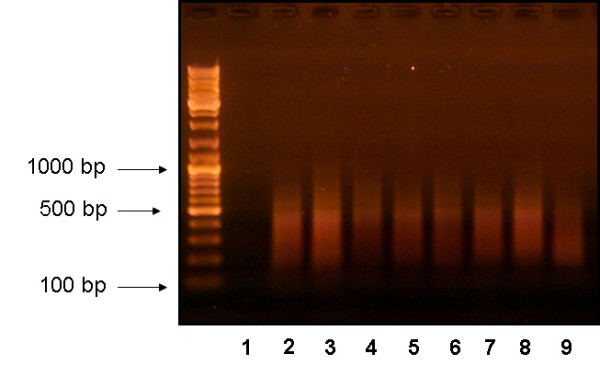
**DOP PCR results of the nanodissected chromosome**. The nanodissected chromosome 1 (lanes 7 and 8), the single nanodissected chromatid of chromosome 3 (lanes 4, 5, 6) and the single microchromosomes nanodissections (lanes 2 and 3) were examined in 1% agarose gel electrophoresis and show a banding pattern between 200 and 600 bp. In lane 1, PCR reaction with no added DNA and in lane 9 the positive control (1 μg/μL Cot-1 DNA).

In the scanning on the whole metaphase plate the chromosome object of nanolitographic dissection has been identified. AFM imaging allows the identification of a pattern of banding as well as a fibrous structure (with diameter of around 50 nm). Structural protrusions along the chromosome correspond to the "G-positive" bands thus making the region to be dissected recognizable with a topographical banding [10]. The band (1p)(p22-p23), that results duplicated in one of the two homologous chromosomes, has been selected in the unduplicated homologue to be dissected in order to produce a probe for the FISH. The chromatid band cht del(3)(q2.10) that results deleted in both chromosomes has been selected to be dissected (Figure 1) and the probe generated. The aim was to show the duplication with molecular methods and to confirm the ability to identify a single chromatid band with the topographical banding. A microchromosome has been likewise selected in order to show its univocal recognizability with hybridization molecular methods, given the non univocal recognizability with traditional cytogenetic methods (Figure 2). Here, we show that DOP-PCR can be applied to a single unstained chromosome or a single chromosomal region without topoisomerase treatment normally used in the experiments of chromatin dissection. The results of the DOP PCR performed with the nanodissected chromosome 1, the single nanodissected chromatid of chromosome 3 and the single microchromosomes nanodissections were examined in 1% agarose gel electrophoresis and show a banding pattern between 200 and 600 bp (Figure 3). The template DNA concentration was comprised from 1 mg/ml and 1.5 mg/ml with 260/280 absorbance of 1.7-1.9. The amplified DNA concentrations were determined by quantitative agarose gel electrophoresis and spectrophotometric analysis: for all the samples, the concentrations obtained after DOP PCR were no proportional to the different forces applied (5-10 μN) for the dissections, indicating that the increase in the depth of the dissection and so in the quantity of the extracted DNA do not affect the quantity of the amplified product.

**Figure 4:**
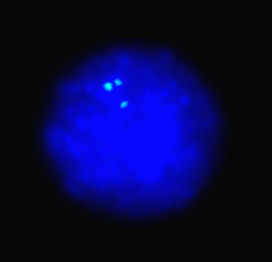
**FISH using DOP-PCR products**. FISH using DOP-PCR products of the nanodissected duplication (1p)(p22-p23). Hybridization of the biotinylated probe DNA is detected with FITC-avidin (green signals). Chromosomes are counterstained with DAPI (blue). The tree signals show that the band (1p)(p22-p23) results duplicated in one of the two homologous chromosomes.

The band specific probe of duplication (1p)(p22-p23) is generated with Biotin-11-dUTP and applied to interphase nuclei (Figure 4). The fluorescent signals were as bright and clear as commercial probes. The probe of single nanodissected chromatid of chromosome 3 is hybridized in interphase nuclei in two distinct spots.

**Figure 5:**
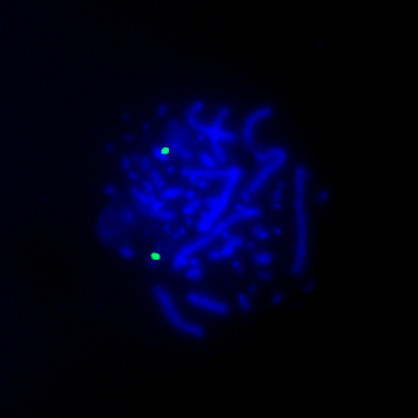
**FISH using DOP-PCR products**. FISH using DOP-PCR products of the nanodissected microchromosome. Hybridization of the biotinylated probe DNA is detected with FITC-avidin (green signals). Chromosomes are counterstained with DAPI (blue). By DOP-PCR of single nanodissected chicken microchromosome, we have generated a chromosome painting probe.

**Figure 6:**
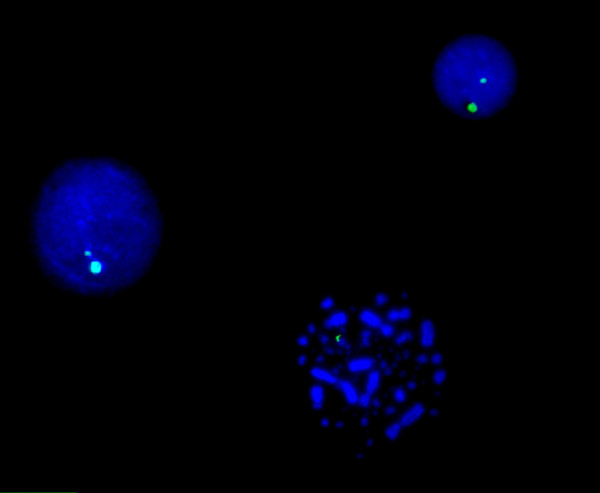
**FISH using DOP-PCR products**. FISH using DOP-PCR products of the nanodissected microchromosome. Hybridization of the biotinylated probe DNA is detected with FITC-avidin (green signals). Chromosomes and nuclei are counterstained with DAPI (blue). We apply FISH technology as a rapid method for detection of MICs aneuploidy as is clear from the only signal in metaphase.

In Figure 5 FISH using DOP-PCR products of the nanodissected microchromosomes is shown. By DOP-PCR of single nanodissected chicken MICs, we have generated a chromosome painting probe (Figure 5). We apply FISH technology as a rapid method for detection of MICs aneuploidy (Figure 6). The presence of dual signals in the nuclei and the single spot in metaphase is explained as somatic mosaicism. About 40% of interphasic nuclei and/or metaphase scored shows aneuploidy. The level of somatic mosaicism directly contributes to carcinogenesis by interfering with the normal division of cells.

Conventional fluorescence microscope make it possible to observe several Kbp DNA probes in metaphase and it remains impossible to observe probes having length shorter than 1 Kbp without several stages of signal amplification. Our probes have a length between 200 and 600 bp. The related fluorescent signal in metaphase is identifiable, with a conventional fluorescence microscope, only for MICs characterized by repeated sequences that allowed repeated hybridization of our probes, thus overcoming the resolution limits of conventional microscopy. The probes hybridization obtained from chromosome 1 and chromatid regions of chromosome 3 is confirmed by the fluorescent signal in interphasic nuclei where the DNA appears in a more loose form which allows the visualization by mean of conventional fluorescence microscopes.

## Discussion

This work shows clearly that DNA can be mechanically extracted by the AFM for subsequent use in molecular cytogenetics. To date, various investigators have applied the AFM to the dissection of chromosomes at different regions [13]. We introduce nanolithography on chromosomes surface. In our laboratory we have reduced the 17 μN applied force for the achievement of hybridization probes used in Iwabuchi's work and co-authors [14], until 5 μN, minimum value successfully used by us. The applied forces are comparable to those used by Oberringer and co-workers [15]. In addition we have remarkably reduced the size of the dissected fragment from 1 μm obtained by Yamanaka and co-authors [16] reaching a length of 400 nm.

Our experiments have clearly shown that dissected DNA can subsequently be used as material for PCR amplification and labeling to generate single-cell genetic probe for FISH analysis. By means of a conventional fluorescence microscope it is possible to observe DNA probes in metaphase having a length of several Kbp and it remains impossible to observe probes having length inferior to 1 Kbp without several stages of signal amplification. Our probes have a length from 200 and 600 bp. The hybridization obtained by mean of the chromosome 1 and chromatid regions of chromosome 3 generated probes is confirmed by the fluorescent signal in interphasic nuclei where the DNA appears in a more looser form which allows the visualization by mean of conventional fluorescence microscopes. As demonstrated by Oberringer and co-workers, Scanning Near-field Optical Microscope (SNOM) is presently necessary for the optical visualization of probes with dimensions comparable to those obtained by us [15].

Moreover, AFM and other new technologies such as SNOM, may allow in the future more exhaustive examinations of metaphase chromosomes and associations between chromosomal aberrations and diseases at a nanoscale level. SNOM/AFM, in fact, can simultaneously obtain topographic and fluorescent images with nanometer-scale resolution. The application of AFM can be a useful horizons for human cytogenetic studies such as in cases of recombination at low copy repeats resulting in tiny deletion/duplication of DNA (Prader-Willi and Angelman syndromes or Charcot-Marie-Tooth disease). A further limitation of classical cytogenetic and largely molecular techniques is the lack of capacity to asses clinically important characteristics of the target cells. In patient with multiple myeloma, for example, routine FISH assessment may yield normal results owing to the low percentage of diseased plasma cells. Thus, a method to generate single-cell genetic probes is needed in this type of study.

Many advantages are attributable to the use of AFM techniques. These include the high sensibility, the short time required for the application and the low quantity of manipulation or chemical treatments that can affect the structure of chromosomes. Finally it can not be undervalued the low cost of the application in comparison to traditional techniques. Recent advances in AFM technology have improved the resolution using a liquid environment. It will be interesting to continue our studies using these new opportunities in conditions close to chromosome physiological state.

## Conclusions

This work demonstrates how it is possible to generate genetic probes for a single specific cell starting from a small region of chromosome or chromatid dissected by an AFM tip. We have thus achieved a real metaphase chromosome nanolitography. This strategy opens the way for new applications in research and diagnostic cytogenetics, evolutionary studies or physical mapping of the genome. Small amounts of DNA from specific and recognizable sites can be amplified and biotinylated using standard molecular biology techniques to be hybridized to metaphase plates and interphase nuclei. Implementing this method using scanning near field optical microscopy for fluorescence imaging, can definitely improve the resolution presently limited by optical microscopy thus achieving the study of specific genomic region labeled with only few dye molecules.

## Methods

### Cell culture and chromosome suspension preparation

MDCC-MSB1 cells were cultured in RPMI medium, supplemented with 10% heat-inactivated foetal calf serum (FCS), 100 μg/ml penicillin, 100 μg/ml streptomycin at 37°C in 5% CO_2_. The reagents for cell culture were purchased from Laboratoires Eurobio (France). For chromosome suspension preparation during the logarithmic growth phase, Colchicine (Sigma, final concentration of 0.05 μg/ml) was added to the cultures that were then mixed gently and incubated at 37°C for 3 h prior to experiments. The cells were collected, centrifuged for 10 min at 1200 rpm, and the supernatant discarded. The pellet was gently overlain with 10 ml of phosphate buffer solution (PBS, pH 7.4) three times and subjected to hypotonic treatment (0.45% sodium citrate) for 10 min before being fixed dropwise in 10 ml cold freshly made fixative, methanol/acetic acid (3:1). The chromosome suspension was then stored in fixative at 4°C for at least 12 h.

### Slide preparation and topographic banding

The chromosome suspension was centrifuged at 1200 rpm for 10 min, the supernatant discarded, and the pellet was resuspended in cold freshly made fixative again to see it cleaned up. The last pellet was resuspended in 0.8/1.0 ml of fixative. Chromosomes were spread on a frosted microscope slide previously washed in fixative diluted in water and put at -20°C in distilled water for at least 1 h. Slides were checked under a phase-contrast microscope to ensure that the cell density was correct, and that there were sufficient well-spread, cytoplasm-free mitoses. The slides were finally air-dried. For GTG Banding (Giemsa banding after Trypsin treatment), after aging for three days, slides were placed in 0.1% trypsin solution for 20 seconds, rinsed with 70% ethanol, washed with water and stained in 5% Giemsa's solution in pH 6.8 PBS.

For topographic banding with the AFM, the slides were washed in 2 × SSC (0.15 M NaCl, 0.015 M sodium Citrate) for 10 min. Chromosomes were treated with RNase A (Boehringer) stock solution (20 mg/ml) diluted 1:200 in 2 × SSC and incubated for 40 min at 37°C. The slides were then washed in 2 × SSC for 5 min three times, shaking at room temperature. For protein digestion 10 μl of Pepsin (Sigma, 100 mg/ml) were added to 100 ml of 0.01 M HCl. The slides were incubated in pepsin solution for 5 min at 37°C and washed in pH 7.4 PBS buffer for 10 min at room temperature. Finally, the slides were dehydrated in an alcohol series (30-50-70-90-99% of Ethanol) prior to AFM analysis (NT-MDT SMENA on Olympus IX71 Inverted Fluorescence Microscope). To identify the Topographic Bands, several cross-line profiles through the long axis of the chromosomes were measured and compared to the GTG profiles. The ridges of the chromosomes cross-line profiles were associated with the GTG+ bands and the grooves with the GTG- bands.

### Atomic Force Microscopy Nanolithography

The nanodissection experiments were carried on by a Smena AFM (NT-MDT, Zelenograd, Russia) operated both in intermittent contact and in contact mode. The cantilever used were NSG10 (NT-MDT, Zelenograd, Russia), with a resonance frequency of 140-390 KHz and a nominal spring constant of 37.6 N/m. The AFM used for these experiments is coupled with an inverted optical microscope Olympus IX70 (Olympus, Japan) that allows finding the proper metaphase nuclei and to position the cantilever on the chromosomes that compose it. Intermittent contact imaging allows identitying all the chromosomes in the chosen metaphase and locating the proper heterochromatin/euchromatin regions, identified by topographic banding, to perform the nanodissection experiments. Upon localization of the chromosome region to be dissected the AFM microscope is switched in Contact Nanolithography Mode and the probe is scanned at high force (5-10 μN) several times (4 to 6) for few lines (up to 8) perpendicularly to the chromatide, this procedure allows the tip to remove the portion of chromatine corresponding to the scanned lines. The cantilever is then lifted immediatly and stored in the recovery buffer for the further DNA amplification. To verify the lithographic dissection, imaging is performed on the same chromosome that underwent the procedure to see the effective missing portion.

### Degenerate Oligonucleotide-primed Polymerase Chain Reaction (DOP-PCR)

DOP-PCR of nanodissected chromosomes was performed in a MyCycler ™ Thermal Cycler (BIORAD Laboratoires, USA). The premixed double concentrated DOP-PCR master mix contains 3 U AccuSure DNA Polymerase (Bioline USA Inc.) in 120 mM Tris-HCl, 60 mM (NH_4_)_2_SO_4_, 20 mM KCl, 4 mM MgSO_4_, pH 8.3, MgCl_2 _3 mM, Brij 35 (Sigma) 0.02% (v/v), dNTPs 0.4 mM. The DOP-PCR reactions was directly performed in the sterile tubes containing the dissected chromosome fragments adhered to the AFM tip in Tris-HCl 40 mM pH 8.3, MgCl_2 _20 mM, NaCl 50 mM. In every tube were added 2 μM 6 MW primer (5'CCGACTCGAGNNNNNNATGTGG3', MWG Eurofins Operon, Germany), the DOP-PCR master mix and sterile water to a final volume of 50 μl. Primary amplification was performed with the following cycling parameters: initial denaturation at 95°C for 5 min, 5 low stringency cycles of 94°C for 1 min, 30°C for 1.5 min, 3 min transition of 30°to 72°C and 72°C for 3 min, followed by 35 high stringency cycles of 94°C for 1 min, 62°C for 1 min, 72°C for 1 min and a final extension of 7 min at 72°C. The PCR products were analyzed by electrophoretic separation on 1% agarose gel. 5 μl of the primary PCR products were labeled with Biotin-11-dUTP ( Fermentas) in a secondary PCR reaction. The 50 μl labelling reaction contained 1.25 U Taq DNA Polymerase (Fermentas) in 10 mM Tris-HCl pH 8.8, 50 mM KCl, 0.08% Nonidet P40, 2 mM MgCl_2_, 0.2 mM dATP, dCTP and dGTP, 100 μM dTTP and 80 μM Biotin-11-dUTP (Fermentas). Cycling parameters were: initial denaturation at 94°C for 3 min, 20 cycles of 94°C for 1 min, 56°for 1 min, 72°for 30 sec and final extension for 3 min. Labeled products were recovered by ethanol precipitation and 500 ng of biotinylated products with 100 fold excess of Chicken Cot-1 DNA were resuspended in hybridization solution (50% deionized formamide, 2X SSC, 10% dextran sulfate).

### Chicken Cot-1 DNA and Probe preparation

Chicken Cot-1 DNA (not commercially available) is the repetitive sequence of Chicken genomic DNA used as a competitor to inhibit hybridization of repeats present within DNA probes. Total genomic DNA was isolated and boiled for 90 min to obtain fragments size of 300-600 bp. The fragmented DNA (1 mg/ml) was denatured in 0.3 M NaCl at 95°C for 10 min and then allowed to reanneal at 65°C for 6 min. An equal volume of ice-cold 2× S1 nuclease buffer and S1 nuclease (Fermentas) was added and incubated at 37°C for 30 min. An equal volume of 25:24:1 phenol:chloroform:isoamyl alcohol was added and mixed well inverting the tube for 10-15 times and then centrifuged for 10 min at 5000 rpm at room temperature. The supernatant was transferred into a new tube and a equal volume of chloroform was added, mixed well and centrifuged for 10 min at 5000 rpm at RT. The supernatant was transferred into a new tube and 1/12^th ^volume of 3 M NaCl was added, mixed well, and 2.5 volume of 100% ethanol was then added and incubated at -20°C overnight. The tube was centrifuged at 5000 rpm for 30 min and the pellet wash whit 70% of ethanol, air-dried and resuspended in distilled water. The Cot-1 DNA were analyzed by electrophoretic separation on 1% agarose gel and concentration adjusted to 1 μg/μl. The DOP-PCR labelled probes were dissolved in 50% formamide, 10% dextran sulphate and 2 × SSC to a final concentration of 50 ng/μl with a 100 fold excess of chicken Cot-1 DNA.

### Fluorescence in situ hybridization (FISH)

The slide-mounted cells were placed for 2 min in a denaturing solution (70% deionized formamide/2 × SSC, pH 7.0) at 70°C in a Coplin jar and then rinsed for 2 min in ice-cold 70% ethanol to stop the denaturation. The dehydration was continued by incubating slide for 2 min each at room temperature in 80-95-100% ethanol. The slides were finally air-dried. 20 μL of hybridization solution is denatured at 75°C for 5 min, applied to slide and covered with a 22-mm^2 ^coverslip. Hybridization was at 37°C overnight in a moist chamber. Slides were washed in 50 ml of 50% formamide/2 × SSC at 39°C for 15 min, 2 × SSC at 39°C for 15 min, 1 × SSC at room temperature for 5 min and allowed to equilibrate 5 min in 4 × SSC at room temperature. 50 μL of biotin detection solution (Avidin-FITC, Vector Laboratories) was applied and incubated 45 min in a aluminium foil-wrapped moist chamber at 37°C. The slides were sequentially soak in aluminium foil wrapped Coplin jars containing room temperature 4 × SSC, 0.1% Triton X-100/4 × SSC, and 4 × SSC 10 min in each solution. The slide was counterstained with DAPI (4,6-diamidino-2-phenylindole dihydrochloride). FISH signals were captured by a Zeiss Axioplan 2 fluorescence microscope with epi-illumination and filter set appropriate for the fluorochrome used.

## Competing interests

The authors declare that they have no competing interests.

## Authors' contributions

SDB has made substantial contributions to conception and design, acquisition, collection, analysis, and interpretation of data; has drafted the manuscript. MFG has prepared cells, LDL has performed the DOP-PCR experiments, FV has made substantial contributions for the use of Atomic Force Microscope. FB supported in the AFM experiments and in the critical revision. AP and DB were been involved in drafting and revising the manuscript critically for important intellectual content. All authors read and approved the final manuscript.
